# The sensitivity of the QuantiFERON^®^-TB Gold Plus assay in Zambian adults with active tuberculosis

**DOI:** 10.5588/ijtld.16.0764

**Published:** 2017-06

**Authors:** L. Telisinghe, M. Amofa-Sekyi, K. Maluzi, D. Kaluba-Milimo, M. Cheeba-Lengwe, K. Chiwele, B. Kosloff, S. Floyd, S-L. Bailey, H. Ayles

**Affiliations:** * School of Social and Community Medicine, University of Bristol, Bristol, UK; † Zambart, Ridgeway Campus, Lusaka, Zambia; ‡ Faculty of Infectious and Tropical Diseases, London School of Hygiene & Tropical Medicine, London, UK; § Faculty of Epidemiology and Population Health, London School of Hygiene & Tropical Medicine, London, UK

**Keywords:** interferon-gamma release assays, latent tuberculous infection, validity

## Abstract

**SETTING AND OBJECTIVE:**

To investigate the sensitivity of the new interferon-gamma release assay (IGRA), QuantiFERON^®^-TB Gold Plus (QFT-Plus), for active TB (used as a surrogate for latent tuberculous infection) in a Zambian TB clinic.

**DESIGN:**

Consecutive smear or Xpert^®^ MTB/RIF-positive adult (age ⩾18 years) pulmonary TB patients were recruited between June 2015 and March 2016. Venous blood was tested using QFT-Plus. The sensitivity was defined as the number positive divided by the total number tested. Using logistic regression, factors associated with positive QFT-Plus results were explored.

**RESULTS:**

Of 108 patients (median age 32 years, interquartile range 27–38; 73% male; 63% human immunodeficiency virus [HIV] positive), 90 were QFT-Plus-positive, 11 were negative and seven had indeterminate results; sensitivity was 83% (95%CI 75–90). There was no difference in sensitivity by HIV status (HIV-positive 85%, 95%CI 75–93; *n* = 68 vs. HIV-negative 80%, 95%CI 64–91; *n* = 40; *P* = 0.59). In models adjusted for age alone, CD4 cell count <100 cells/μl (OR 0.15, 95%CI 0.02–0.96; *P*=0.05) and body mass index <18.5 kg/m^2^ (OR 0.27, 95%CI 0.08–0.91; *P* = 0.02) were associated with decreased odds of positive QFT-Plus results.

**CONCLUSION:**

Overall, the sensitivity of QFT-Plus is similar to that of the tuberculin skin test and other IGRAs. While overall sensitivity is not affected by HIV status, QFT-Plus sensitivity was lower among people living with HIV/acquired immune-deficiency syndrome with severe immunosuppression.

## RESUME

**CONTEXTE et OBJECTIFS:**

Examiner la sensibilité du nouveau test de libération de l'interféron gamma (IGRA), QuantiFERON^®^-TB Gold Plus (QFT-Plus), pour la tuberculose (TB) active (utilisée comme substitut de l'infection tuberculeuse latente) dans une centre TB de Zambie.

**SCHÉMA:**

Des patients adultes consécutifs (âge ⩾18 ans) atteints de TB pulmonaire à frottis positif ou Xpert positif ont été recrutés entre juin 2015 et mars 2016. Leur sang veineux a été testé avec QFT-Plus. La sensibilité a été le nombre de résultats positifs sur le total testé. Une régression logistique a permis d'explorer les facteurs associés avec la positivité de QFT-Plus.

**RÉSULTATS:**

Parmi 108 patients (âge médian 32 ans, intervalle interquartile 27–38 ; 73% d'hommes et 63% positifs au virus de l'immunodéficience humaine [VIH]), il y a eu 90 résultats de QFT-Plus positifs, 11 négatifs et sept indéterminés, soit une sensibilité de 83% (IC95% 75–90). Il n'y a pas eu de différence de sensibilité en fonction du statut VIH (VIH positifs 85% ; IC95% 75–93 ; *n*=68 et VIH négatifs 80% ; IC95% 64–91 ; *n*=40 ; *P* = 0,59). Dans les modèles ajustés sur l'âge seulement, la numération des CD4 <100 cellules/μl (OR 0,15 ; IC95% 0,02–0,96 ; *P* = 0,05) et l'indice de masse corporelle <18,5 kg/m^2^ (OR 0,27 ; IC95% 0,08–0,91 ; *P* = 0,02) ont été associés avec une diminution de la probabilité de résultats positifs de QFT-Plus.

**CONCLUSION:**

La sensibilité d'ensemble de QFT-Plus est similaireà celle du test cutanéà la tuberculine et à d'autres IGRA. Si la sensibilité d'ensemble n'est pas affectée par le statut VIH, elle a cependantété plus faible parmi les personnes vivant avec le VIH/SIDA (syndrome d'immunodéficience acquise) atteints d'immunosuppression grave.

## RESUMEN

**MARCO DE REFERENCIA Y OBJETIVOS:**

Investigar la sensibilidad de una nueva prueba de liberación de interferón-gama (IGRA), la QuantiFERON^®^-TB Gold Plus (QFT-Plus), en la detección de la tuberculosis (TB) activa (utilizada como indicador indirecto de la infección tuberculosa latente) en un consultorio de TB en Zambia.

**MÉTODO:**

Se incluyeron en estudio los pacientes adultos (a partir de los 18 años de edad) consecutivos con diagnóstico de TB pulmonar y un resultado positivo de la baciloscopia o de la prueba Xpert^®^ MTB/RIF de junio del 2015 a marzo del 2016. Se practicó la prueba QFT-Plus en una muestra de sangre periférica. La sensibilidad se definió como el número de resultados positivos sobre el total de pruebas realizadas. Mediante un análisis de regresión logística se analizaron los factores asociados con un resultado positivo de la prueba QFT-Plus.

**RESULTADOS:**

De los 108 pacientes (mediana de la edad 32 años; amplitud intercuartílica de 27 a 38; 73% de sexo masculino y 63% positivos frente al virus de la inmunodeficiencia humana [VIH]), 90 obtuvieron un resultado positivo de la prueba QFT-Plus, 11 pacientes un resultado negativo y en 7 casos el resultado fue indefinido; la sensibilidad fue 83% (IC95% de 75 a 90). No se observó ninguna diferencia de sensibilidad en función de la situación frente al VIH (VIH positivos 85% ; IC95% de 75 a 93; *n* = 68 y VIH negativos 80% ; IC95% de 64 a 91; *n* = 40; *P* = 0,59). En los modelos ajustados según la edadúnicamente, los factores asociados con una menor probabilidad de obtener un resultado positivo de la prueba QFT-Plus fueron un recuento de linfocitos CD4 <100 células/μl (OR 0,15; IC95% de 0,02 a 0,96; *P* = 0,05) y un índice de masa corporal <18,5 kg/m^2^ (OR 0,27; IC95% de 0,08 a 0,91; *P* = 0,02).

**CONCLUSIÓN:**

La sensibilidad global de la prueba QFT-Plus es equivalente a la sensibilidad de la reacción tuberculínica y de otras IGRA. Esta sensibilidad no se afecta por la situación frente al VIH, pero fue inferior en las personas con infección por el VIH y síndrome de inmunodeficiencia adquirida que presentaban una inmunodepresión grave.

THE INCREASED RISK of active tuberculosis (TB) among people living with human immunodeficiency virus/acquired immune-deficiency syndrome (PLHIV)^1^,^2^ can be substantially decreased by treating latent tuberculous infection (LTBI) with isoniazid preventive therapy (IPT).^3^ However, there are numerous barriers to IPT use,^4^,^5^ including the lack of a good diagnostic test for LTBI among PLHIV.^6^

Two imperfect but widely used tests for LTBI that measure the host's cell-mediated immune response to Mycobacterium tuberculosis antigens are the tuberculin skin test (TST) and interferon-gamma (IFN-γ) release assays (IGRAs) (QuantiFERON^®^-TB Gold In-Tube [QGIT], QIAGEN, Hilden, Germany; and the T-SPOT^®^.*TB* test, Oxford Immunotec, Abingdon, UK).^6^,^7^ Both tests have poor and comparable sensitivity among PLHIV (TST sensitivity 45% and IGRA sensitivity 60–75%).^7^–^9^

CD4+ T-lymphocytes play a critical role in the immunological control of M. tuberculosis through the secretion of IFN-γ;^10^ QGIT mainly evokes and measures this CD4-mediated response.^11^ There is growing evidence to suggest that CD8+ T-lymphocytes also play a role in M. tuberculosis control through the production of IFN-γ,^10^,^12^–^14^ and M. tuberculosis-specific CD8+ T-lymphocytes have been identified in subjects with LTBI and active TB.^14^,^15^ The QGIT has thus now been modified to include two antigen tubes—TB1, which contains a long peptide cocktail aimed at stimulating CD4+ T-lymphocytes, and TB2, which contains an additional shorter peptide cocktail aimed at stimulating CD8+ T-lymphocytes in addition to CD4+ T-lymphocytes^16^—now called QuantiFERON^®^-TB Gold Plus (QFT-Plus). The antigen TB 7.7, found in QGIT, has been removed.^16^ This approach, which negates the dependence on CD4-mediated responses alone, could potentially increase the test's validity for LTBI among PLHIV.

We conducted a study in Lusaka, Zambia, a setting with high TB and HIV prevalence, to determine the sensitivity of QFT-Plus. As a gold standard for LTBI does not exist, active TB was used as a surrogate, as patients with active TB are infected with M. tuberculosis.

## METHODS

### Study population and procedures

The study was based in a government health facility. All patients with suspected pulmonary TB underwent sputum testing with either fluorescent microscopy or the Xpert^®^ MTB/RIF assay (Cepheid, Sunnyvale, CA, USA); pulmonary TB was defined as at least one positive sputum sample on either smear or Xpert. Culture is not routinely performed. HIV counselling and testing according to national guidelines is routinely offered to all patients investigated and/or treated for TB. For HIV testing, sequential blood-based rapid antibody tests with Determine^®^HIV-1/2 Antibody (Alere, Waltham, MA, USA) is used first, which, if reactive, is followed by the Uni-Gold^™^ Recombigen HIV-1/2 Antibody test (Trinity Biotech, Bray, Ireland).^17^

Between June 2015 and March 2016, consecutive adult (age ⩾18 years) pulmonary TB patients who were ⩽2 days from starting anti-tuberculosis treatment were recruited for the study. Written voluntary informed consent was provided by all study participants. Information on demographics (age, sex), TB and HIV diagnosis and physical measurements (height and weight) were obtained. Body mass index (BMI), defined as (weight in kg)/(height in m)^2^, was classed into standard categories (<18.5 underweight, 18.5–24.9 normal weight, 25–29.9 overweight, ⩾30 obese).^18^

Venous blood for testing with QFT-Plus was collected into lithium heparin tubes, stored at room temperature and transported within 8 h to the laboratory for processing according to the manufacturer's specification.^16^ Venous blood was also collected for CD4 cell count estimation if patients were HIV-co-infected.

### Laboratory procedures

Blood (1 ml) was transferred to each of the four QFT-Plus tubes (nil [background], TB1 [CD4-mediated response], TB2 [CD4- and CD8-mediated response] and mitogen [positive control]), and incubated at 37°C for 16–24 h within 16 h of collection. Following incubation, the samples were returned to ambient room temperature and centrifuged at 2000–3000 relative centrifugal force for 15 min. Plasma was extracted and transferred to a −80°C freezer. Enzyme-linked immunosorbent assay (ELISA) was performed, following re-centrifugation of plasma using standard test kits. The ELISA plate was read at wavelengths of 450 nm and 650 nm. Data were transferred to the QFT-Plus analysis software to calculate results. Positive results were defined according to the manufacturer's threshold (⩾0.35 international unit [IU]/ml); a positive result on either antigen tube, TB1 or TB2, was considered positive. The upper limit for quantifying IFN-γ concentrations was 10 IU/ml. T-lymphocyte estimation (CD4^+^, CD8^+^ and CD3^+^) was performed within 48 h of blood collection using BD FACSCount reagents and a BD FACSCount flow-cytometer (BD, Sparks, MD, USA), according to the manufacturer's specification. The upper limit for CD8 cell count measurement was 2000 cells/μl.

### Patient follow-up

To explore whether negative/indeterminate QFT-Plus results were due to TB and/or HIV-associated immunosuppression, which should improve upon treatment, patients with negative or indeterminate QFT-Plus results at enrolment underwent repeat testing with QFT-Plus at ~1–2 months post-enrolment.

### Statistical analysis

The target sample size was 100 pulmonary TB patients, aiming to determine an overall QFT-Plus sensitivity of 74% (based on QGIT sensitivity in this population^11^) with a precision of ±9% assuming 95% confidence intervals (CIs). A higher sensitivity of 85% would give a precision of ±7%.

Data were analysed using STATA, version 12.0 (StataCorp LP, College Station, TX, USA). Sensitivity was defined as the number of positive results divided by the total number tested at enrolment; the denominator included indeterminate results. To explore factors associated with positive QFT-Plus results, negative and indeterminate QFT-Plus categories were combined to give a binary variable. Logistic regression was used to determine odds ratios (ORs), 95%CIs and *P* values from the likelihood ratio test. Age was considered a forced variable a priori. Due to the small number of negative/indeterminate results, a multivariable analysis could not be undertaken. The analysis was therefore limited to a univariate analysis consisting of base models (each factor adjusted for age) alone. Results are presented following STARD (Standards for Reporting of Diagnostic Accuracy) guidelines.^19^

### Ethics approval

Ethics approval for the study was obtained from the Biomedical Research Ethics Committees of the University of Zambia, Lusaka, Zambia, and the London School of Hygiene & Tropical Medicine, London, UK.

## RESULTS

**Figure 1.**
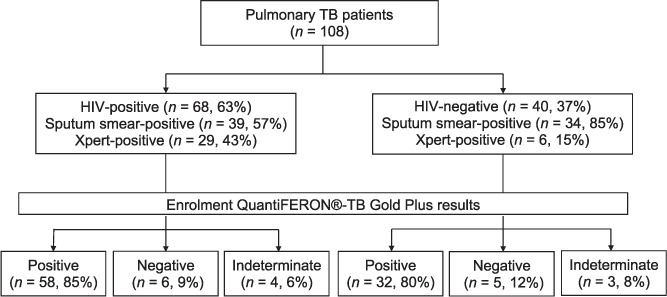
Patients with pulmonary TB enrolled in the study: HIV status, microbiology results and QuantiFERON^®^-TB Gold Plus results. TB = tuberculosis; HIV = human immunodeficiency virus.

A total of 108 pulmonary TB patients were recruited (Figure 1). The median age of the 104 patients with data was 32 years (interquartile range [IQR] 27–38); 76/104 (73%) were male and 104/104 (100%) were of Black African ethnicity (Table 1). Over half (55/104, 53%) had a BMI <18.5 kg/m^2^ and 68/108 (63%) were HIV-co-infected.

**Table 1:**
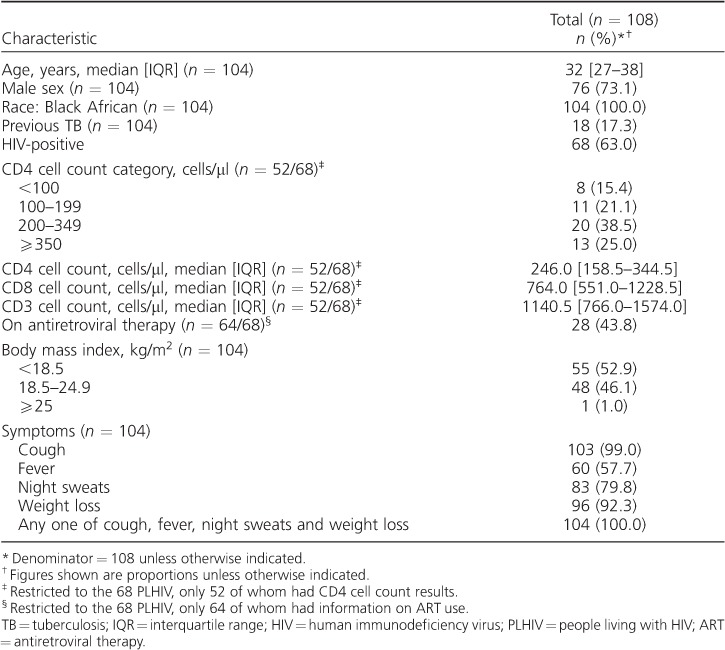
Characteristics of pulmonary TB patients (*n* = 108)

### QFT-Plus sensitivity

Ninety patients had positive QFT-Plus results, 11 were negative and seven had indeterminate results at enrolment, giving an overall sensitivity of 83% (95%CI 75–90). The median TB1 and TB2 concentrations, minus the nil background IFN-γ concentrations (TB1-nil and TB2-nil), were respectively 2.00 (IQR 0.45–7.50) and 3.81 (IQR 0.59–10.00) IU/ml (*P* < 0.001) (Appendix Figure A).^*^ When the overall results were stratified by antigen tubes, 6/24 (25%) negative/indeterminate results on TB1 were positive on TB2 (Table 2).

**Table 2:**
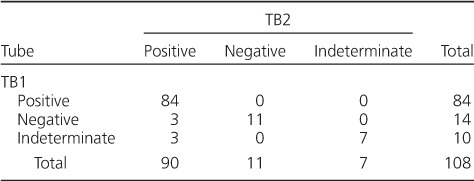
Overall test results (positive, negative, indeterminate) of pulmonary tuberculosis patients by QuantiFERON^®^-TB Gold Plus tube (*n* = 108)

The sensitivity of QFT-Plus among PLHIV was similar to that among those who were HIV-negative (85%, 95%CI 75–93, and 80%, 95%CI 64–91, respectively, *P* = 0.59; Table 3). Among PLHIV, sensitivity was lower with CD4 cell counts <100 cells/μl than with CD4 ⩾100 cells/μl (50%, 95%CI 16–84 and 89%, 95%CI 75–96, respectively, *P* = 0.02). QFT-Plus sensitivity was lower in those who were underweight than in those who were normal or overweight (76%, 95%CI 63–87 and 92%, 95%CI 80–98, respectively, *P* = 0.04).

**Table 3:**
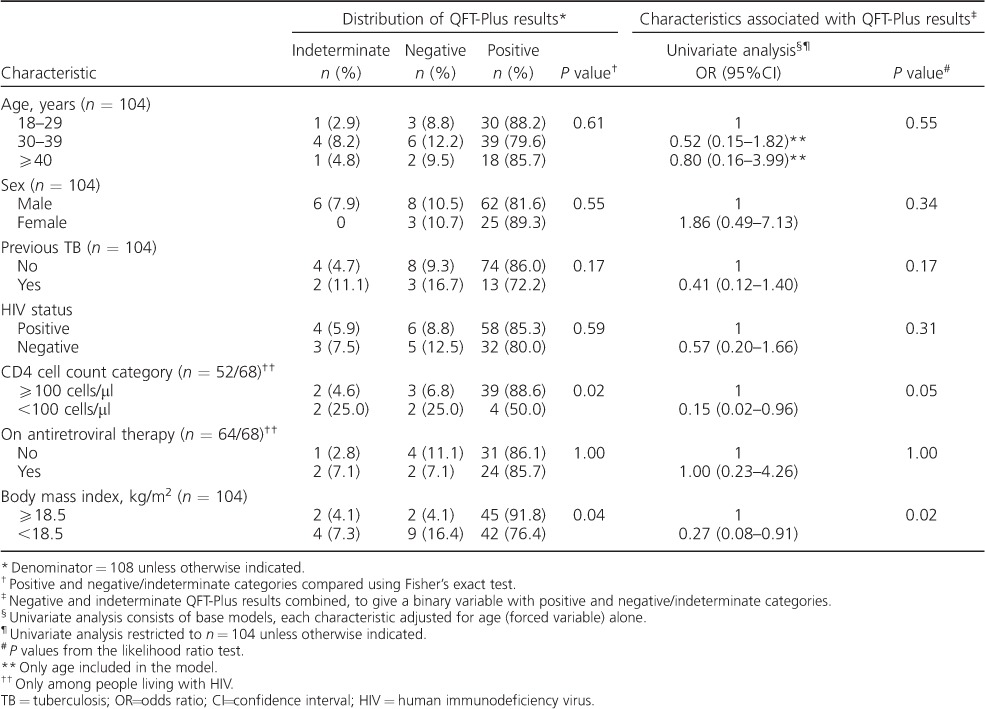
Distribution of QuantiFERON^®^-TB Gold Plus results by patient characteristics and univariate logistic regression analysis of factors associated with positive QFT-Plus results in pulmonary TB patients (*n* = 108)

**Figure 2.**
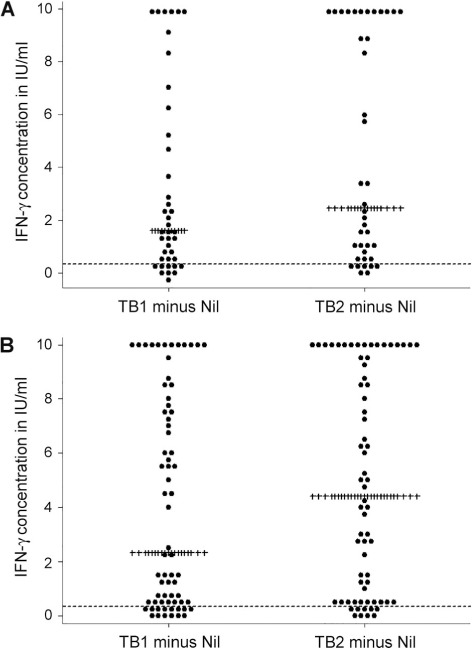
Dot plot of TB1 and TB2, minus the nil background IFN-γ concentration among pulmonary TB patients tested using the QuantiFERON^®^-TB Gold Plus assay, stratified by HIV status (*n* = 108 with known HIV status). **A)** HIV-negative pulmonary TB patients (*n* = 40); **B)** HIV-positive pulmonary TB patients (*n* = 68). Each dot represents the TB1 minus the nil background IFN-γ concentration (on the left) and the TB2 minus the nil background IFN-γ concentrations (on the right), per person. ------ = IFN-γ concentration of 0.35 IU/ml; ++++ = median IFN-γ concentration in IU/ml. IU = international unit; IFN-γ = interferon-gamma; TB = tuberculosis; HIV = human immunodeficiency virus.

The median IFN-γ concentration was higher in TB2 than in TB1, irrespective of HIV status (Figure 2). The median TB1-nil and TB2-nil concentrations among those who were HIV-negative was respectively 1.61 (IQR 0.43–5.74) and 2.47 IU/ml (IQR 0.67–10.00), i.e., the difference in median concentration was 0.86 IU/ml (*P* < 0.001). Among PLHIV it was respectively 2.32 (IQR 0.45–7.87) and 4.42 IU/ml (IQR 0.58–9.77), i.e., the difference in median concentration was 2.1 IU/ml (*P* < 0.001). Among those who were HIV-negative, 2/10 (20%) negative/indeterminate results on TB1 were positive on TB2 (Appendix Table A.1). Among PLHIV, 4/14 (29%) patients with negative/indeterminate results on TB1 were positive on TB2 (Appendix Table A.2). The proportion positive on TB1 and TB2 did not vary by HIV status; respectively 30/40 (75%) and 54/68 (79%) HIV-negative individuals and PLHIV were positive on TB1 (*P* = 0.88), and respectively 32/40 (80%) and 58/68 (85%) were positive on TB2 (*P* = 0.66). When indeterminates were excluded, the overall QFT-Plus sensitivity was 89% (95%CI 81–94, *n* = 90/101; Appendix Table A.3).

### Negative/indeterminate enrolment QFT-Plus results

The characteristics of 18 patients who were QFT-Plus-negative or indeterminate at enrolment are shown separately in Appendix Table A.4. The median age of these patients was 33 years (IQR 30–37); 14/17 (82%) were male and 17/17 (100%) reported at least one of cough, fever, night sweats or unintentional weight loss. Most (13/17, 76%) were underweight. In total, 10/18 (56%) were HIV-positive; the median CD4 and CD8 cell counts of 9 patients with results were respectively 187 (IQR 37–209) and 669 cells/μl (IQR 424–1289). The median TB1-nil and TB2-nil concentrations at enrolment were respectively 0.14 (IQR 0.03–0.27) and 0.24 IU/ml (IQR 0.08–0.32) (Appendix Table A.5).

On univariate analysis (Table 3), CD4 cell count <100 cells/μl (OR 0.15, 95%CI 0.02–0.96, *P* = 0.05) and being underweight (OR 0.27, 95%CI 0.08–0.91, *P* = 0.02) were associated with decreased odds of positive QFT-Plus results.

Of 18 patients with negative/indeterminate enrolment QFT-Plus results, seven (39%) underwent testing at 1–2 months post-enrolment, five of whom (71%) were positive on repeated testing (4/6, 67%, with negative and 1/1, 100%, with indeterminate enrolment results; Appendix Table A.5). The median TB1-nil and TB2-nil concentrations at enrolment and 1–2 months post-enrolment for two patients who were not positive on repeat testing were respectively 0.03 (IQR −0.02 to 0.08) and 0.10 IU/ml (IQR 0.06–0.14), and 0.08 (IQR 0.06–0.10) and 0.09 IU/ml (IQR 0.05–0.13). Among five patients who were positive on repeat testing, these were respectively 0.20 (IQR 0.16–0.27) and 0.31 IU/ml (IQR 0.25–0.33) at enrolment, and 0.83 (IQR 0.47–1.53) and 0.97 (IQR 0.72–1.21) IU/ml at 1–2 months post-enrolment.

## DISCUSSION

This is the first study to describe the sensitivity of the new QFT-Plus assay for TB in a population with a high prevalence of HIV. The assay's overall sensitivity was maintained, regardless of HIV status. However, sensitivity was lower among PLHIV with severe immunosuppression.

A multicentre study across six European hospitals found a QFT-Plus sensitivity of 86% (102/119; 95%CI 78–91%) for bacteriologically confirmed TB,^20^ which is comparable to our findings. All four patients who were TB-HIV-co-infected had positive QFT-Plus results.^20^ While these numbers are too small to draw any firm conclusions, they corroborate our findings of high QFT-Plus sensitivity among PLHIV. QFT-Plus specificity among low-risk subjects in Europe was 97% (95%CI 92–99).^20^ Similar results have been reported from Germany and Japan, where head-to-head comparisons of QFT-Plus and QGIT among predominantly immunocompetent patients showed similar test sensitivities.^21^,^22^

A study using the same methods as our study was conducted by Raby et al. at the same health facility in 2007 to determine QGIT and TST sensitivity in a similar population.^11^ The proportion of TB-HIV-co-infected patients and the median CD4 cell counts among PLHIV were similar in both studies (Table 4). Overall QFT-Plus sensitivity in our study was similar to QGIT and TST sensitivity; however, unlike QGIT and TST, overall QFT-Plus sensitivity was not affected by HIV status. Studies of QGIT in a number of countries have shown a lower overall assay sensitivity among PLHIV;^9^ our findings are thus contrary to these. While QFT-Plus sensitivity, like QGIT, was lower in PLHIV with CD4 cell counts <100 cells/μl, the point estimate suggests that QFT-Plus sensitivity may be higher in those with CD4 cell counts <100 cells/μl than with QGIT. However, the very small number in the strata precludes any firm conclusions being drawn. Larger studies with direct head-to-head comparisons of QFT-Plus and QGIT among severely immunosuppressed patients are needed to investigate this further.

**Table 4:**
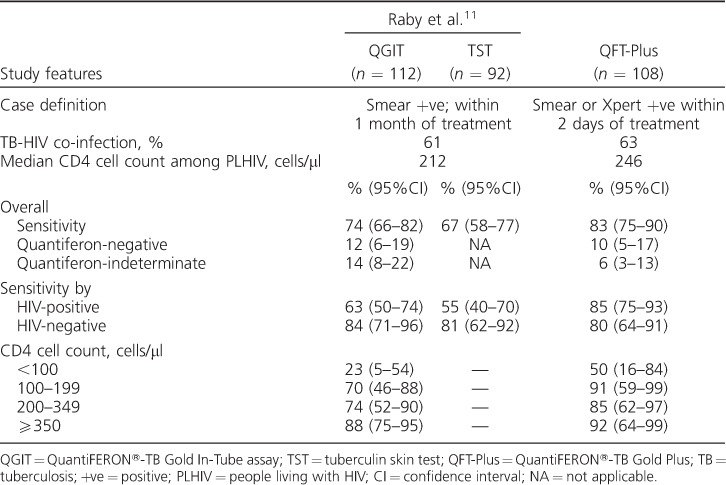
Comparing the performance of QGITassay, the TSTand QFT-Plus among adult (age ⩾18 years) pulmonary TB patients

Like QGIT, the QFT-Plus TB1 tube elicits a CD4-mediated immune response alone. TB1 sensitivity was not affected by HIV status. However, it is not possible to directly infer QGIT sensitivity based on TB1 sensitivity, given the changes that have been made to the antigens and their formulation. Studies with direct head-to-head comparisons of QFT-Plus and QGIT in high HIV prevalence settings are needed to investigate this further.

The higher QFT-Plus sensitivity among PLHIV could have important clinical and policy implications. Given the similar performance but higher costs of IGRAs compared to TST, the World Health Organization recommended the continued use of TST in low- and middle-income countries in 2011.^23^ This stance may need to be re-evaluated in the light of our findings, taking into consideration the reproducibility of the results, direct head-to-head comparisons of QGIT and QFT-Plus, including in severely immunosuppressed patients, costs and logistics. In addition, an ideal test for LTBI should be able to predict LTBI patients who will progress to develop active TB. The predictive value of QFT-Plus for active TB therefore needs to be further investigated.

When compared with QGIT in the study by Raby et al.,^11^ the proportion with indeterminate results on QFT-Plus was lower (Table 4). Indeterminate results may be due to technical errors in sample handling and processing, or immunosuppression of lymphocyte cell lines due to advanced and severe HIV and/or TB.^7^,^16^ Improvements in laboratory procedures over time may therefore partly explain the differences observed. Likewise, earlier diagnosis of HIV and/or TB would result in a shift from indeterminate to positive results. However, TB-HIV-co-infected patients had similar levels of HIV-associated immunosuppression in both studies, suggesting that this is less likely. Finally, targeting both CD8- and CD4-mediated responses by QFT-Plus can increase IFN-γ levels sufficiently, even in the presence of a low IFN-γ response to mitogen, as seen by the three results indeterminate on TB1 which were positive on TB2.

In contrast, negative results indicate an immune response to the positive control, without detectable T-lymphocyte responses to M. tuberculosis-specific antigens. With QFT-Plus, as both CD4- and CD8-mediated IFN-γ responses are targeted, a decrease in negative results was anticipated. However, the proportions with negative QFT-Plus were similar to those of QGIT (Table 4).^11^ The exact mechanism underlying these negative results are unclear. False-positive microbiologicalresults may play a role;however,studies have shown that the specificity of a positive smear or Xpert for M. tuberculosis is high in high TB prevalence settings.^24^–^26^ Representative data on the proportion of smear- or Xpert-positive pulmonary TB patients who are culture-positive for M. tuberculosis in our setting are not available. On univariate analysis, negative/indeterminate QFT-Plus results were associated with a low BMI, which in the context of TB suggests advanced disease that may be associated with TB-specific immune paresis,^27^ resulting in negative responses to M. tuberculosis-specific antigens but positive responses to mitogen. This hypothesis is corroborated by the finding that nearly 70% of negative results at enrolment had converted to positive at 1–2 months following treatment. Studies immunologically characterising negative responses are needed to confirm this. However, the IFN-γ concentrations in a number of these samples were close to the manufacturer's threshold, and may represent fluctuations in values around the threshold. Repeat testing of the samples may therefore have yielded positive results.

Our study has several limitations. It was small and undertaken to explore QFT-Plus sensitivity in a high HIV prevalence setting; CIs around point estimates are therefore wide. Culture was not performed on TB patients. A direct head-to-head comparison of QGIT and QFT-Plus was not undertaken. The results cannot be generalised to children, as only adults were included.

## CONCLUSION

QFT-Plus sensitivity was similar to QGIT and TST sensitivity for pulmonary TB, used as a surrogate for LTBI. While overall sensitivity was not affected by HIV status, the sensitivity was lower among PLHIV with severe immunosuppression. Work is needed to understand the assay's predictive value for active TB.

## Figures and Tables

**Table A.1:**
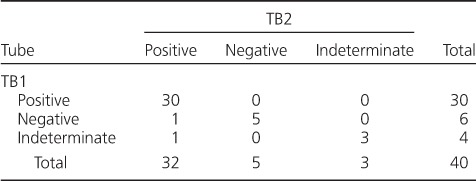
Overall test results (positive, negative, indeterminate) by QuantiFERON^®^-TB Gold Plus tubes for *N* = 40 HIV negative pulmonary TB patients

**Table A.2:**
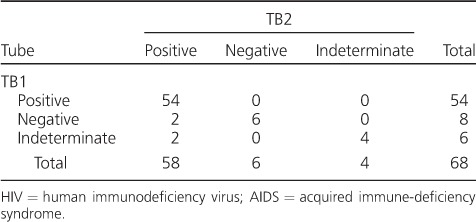
Overall test results (positive, negative, indeterminate) by QuantiFERON^®^-TB Gold Plus tubes for people living with HIV/AIDS with pulmonary TB (*n =* 68)

**Table A.3:**
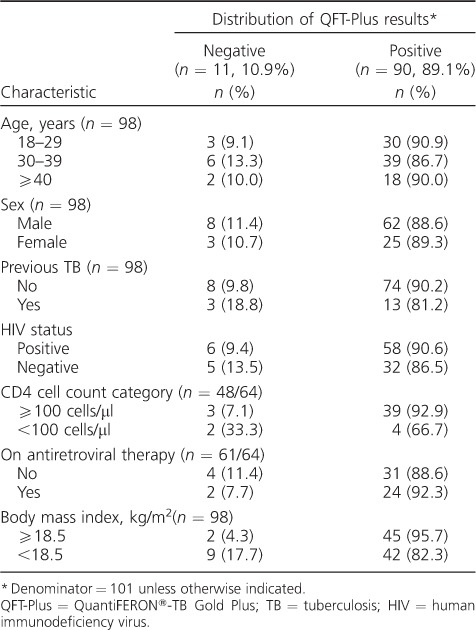
Distribution of QFT-Plus results by patient characteristics for pulmonary TB patients with negative and positive QFT-Plus results (*n* = 101)

**Figure A:**
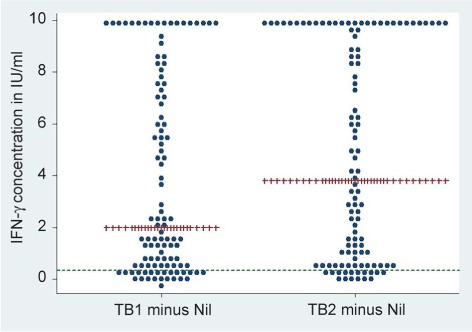
Dot plot of TB1 and TB2, minus the nil background IFN-γ concentration for pulmonary TB patients who underwent QuantiFERON®-TB Gold Plus testing (*n* = 108). Each dot represents the TB1 minus the nil background IFN-γ concentration (on the left) and the TB2 minus the nil background IFN-γ concentrations (on the right), per person. — = IFN-γ concentration = 0.35 IU/ml; ++= median IFN-γ concentration in IU/ml. IFN-γ = interferon-gamma; IU = international unit.

**Table A.4:**
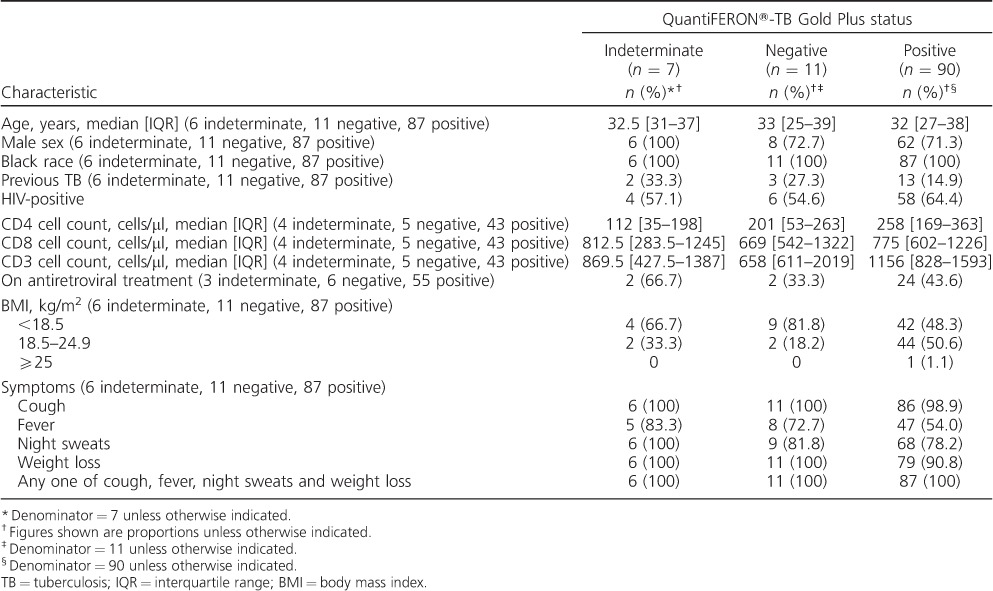
Characteristics of pulmonary TB patients by QuantiFERON^®^-TB Gold Plus status (*n* = 108)

**Table A.5:**
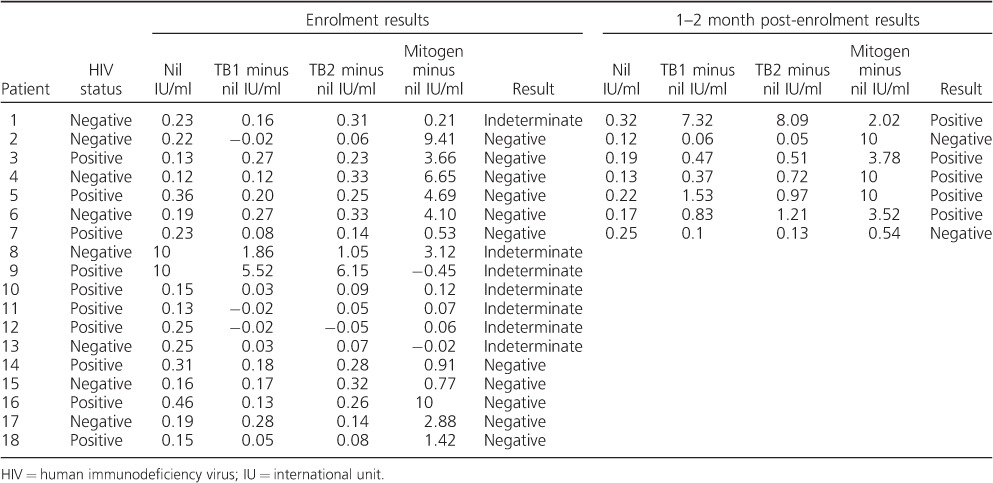
QuantiFERON^®^-TB Gold Plus values at enrolment and 1–2 months post-enrolment, where available, for individuals who were indeterminate or negative at enrolment (*n* = 18)

## Footnotes

^*^ The appendix is available in the online version of this article, at http://www.ingentaconnect.com/content/iuatld/ijtld/2017/00000021/00000006/art00015
